# Omicron KP.3 RBD-Containing Spike mRNA Vaccine Induces Broadly Neutralizing Antibodies with Protection Against SARS-CoV-2 Omicron Infection in Mice

**DOI:** 10.3390/vaccines14010078

**Published:** 2026-01-11

**Authors:** Xiaoqing Guan, Hansam Cho, Shengnan Qian, Qian Liu, Lanying Du

**Affiliations:** Institute for Biomedical Sciences, Georgia State University, Atlanta, GA 30303, USA

**Keywords:** coronavirus, SARS-CoV-2, COVID-19, receptor-binding domain, Omicron subvariants, broadly neutralizing activity, protective efficacy

## Abstract

**Background/Objectives**: Severe acute respiratory syndrome coronavirus 2 (SARS-CoV-2) caused the global COVID-19 pandemic, which led to hundreds of millions of human infections and more than seven million deaths worldwide. Major variants of concern, particularly the Omicron variant and its associated subvariants, can escape the vaccines developed so far to target previous strains/subvariants. Therefore, effective vaccines that broadly neutralize different Omicron subvariants and show good protective efficacy are needed to prevent further spread of Omicron. The spike (S) protein, including its receptor-binding domain (RBD), is a key vaccine target. **Methods**: Here, we designed a unique mRNA vaccine encoding Omicron-KP.3 RBD based on RBD-truncated S protein backbone of an earlier Omicron subvariant EG.5 (KP3 mRNA), and evaluated its stability, immunogenicity, neutralizing activity, and protective efficacy in a mouse model. **Results**: Our data showed that the nucleoside-modified, lipid nanoparticle-encapsulated mRNA vaccine was stable at various temperatures during the period of detection. In addition, the vaccine elicited potent antibody responses with broadly neutralizing activity against multiple Omicron subvariants, including KP.2, KP.3, XEC, and NB.1.8.1. This mRNA vaccine protected immunized transgenic mice from challenge with SARS-CoV-2 Omicron-KP.3. Immune serum also protected against subsequent virus challenge, with the level of protection associating positively with the serum neutralizing antibody titer. **Conclusions**: Taken together, the data presented herein suggest that this newly designed mRNA vaccine has potential against current and future Omicron subvariants.

## 1. Introduction

Severe acute respiratory syndrome coronavirus 2 (SARS-CoV-2) is the causative agent of Coronavirus Disease 2019 (COVID-19). It was first identified in December 2019 and resulted in a global pandemic with a marked impact on public health worldwide [1,2,3,4]. SARS-CoV-2 is highly contagious and transmits very rapidly, leading to devastating levels of illness and deaths; thus, it is different from SARS-CoV and Middle East respiratory syndrome coronavirus (MERS-CoV), although all of these three viruses belong to the beta-genus coronaviruses [5,6,7,8,9,10,11]. Greater than 778.9-million COVID-19 cases, including over 7.1-million deaths, were reported to the World Health Organization (WHO) by 7 December 2025 [12]. In spite of the official ending of the COVID-19 pandemic, SARS-CoV-2 continues to infect humans with deaths, indicating the need for continuous development of safe and effective vaccines to prevent viral infection.

SARS-CoV-2 genome encodes four structural proteins, which include spike (S), membrane, envelope, and nucleocapsid, and these proteins share the same names as those encoded by the respective SARS-CoV and MERS-CoV genomes [5]. Among the above four proteins, the S protein plays a critical role in SARS-CoV-2 infection and its pathogenesis. The S protein contains S1 and S2 subunits, and SARS-CoV-2 infection is processed by initially binding to angiotensin-converting enzyme 2 (ACE2) receptor through the receptor-binding domain (RBD) of the S1 subunit [13,14,15,16]. Viral entry and membrane fusion occur after receptor binding, which are mediated by the S1 and S2 subunits, respectively [17,18,19,20]. Thus, the S protein and its functional RBD fragment are major targets for the development of SARS-CoV-2 vaccines. The RBD-containing S protein has a conformational trimeric structure [15,17]. Accordingly, vaccines potentially forming native trimeric structures would be more preferable to induce favorable immune responses.

Different from SARS-CoV and MERS-CoV, SARS-CoV-2 mutates rapidly. Five major variants of concern (VOCs) have been identified (Alpha, Beta, Gamma, Delta, and Omicron), and there are many variants of interest [21,22,23,24]. Unlike the four VOC strains identified earlier, such as Alpha, Beta, Gamma, and Delta, the Omicron variant contains numerous mutations in the S protein, particularly within the RBD region, leading to the emergence of dozens of Omicron subvariants such as KP.2, KP.3, XEC, and NB.1.8.1 [23,25,26,27]. Notably, vaccines targeting the S protein, especially the RBD fragment, of the original strain or early variants of SARS-CoV-2 have completely lost, or have markedly reduced, neutralizing activity and/or protective efficacy against Omicron subvariants [28,29,30,31]. Therefore, development of vaccines that broadly neutralize different Omicron subvariants and show good protective efficacy is critical to prevent further spread of Omicron.

mRNA technology has become a critical tool for rapid development of much-needed vaccines against various viral pathogens, such as SARS-CoV-2, Zika virus, influenza virus, respiratory syncytial virus, HIV-1, Ebola virus, and Nipah virus, in addition to some cancers [32,33,34,35,36,37]. Unlike traditional vaccines, mRNA vaccines have a strong safety profile and potent protective efficacy, and they are also cost-effective to manufacture and easy to scale up [38]. These properties of mRNA vaccines resulted in fast development and emergency approval of two SARS-CoV-2 mRNA vaccines to stop the COVID-19 pandemic: one from Moderna and one from Pfizer-BioNTech [39,40,41], as well as many other mRNA vaccines that are in clinical trials and/or pre-clinical development [42,43,44,45].

In this study, we generated a unique mRNA vaccine that encodes the RBD of SARS-CoV-2 Omicron-KP.3 subvariant and is built on the conserved backbone region of an RBD-truncated S protein of an earlier Omicron subvariant, EG.5. To promote the formation of a native conformational structure, this vaccine construct was fused to a C-terminal foldon trimeric motif and synthesized for mRNA in the presence of the required elements. The well-characterized, lipid nanoparticle (LNP)-encapsulated KP3 mRNA was further evaluated to assess its broadly neutralizing activity against multiple recent Omicron subvariants and its protective efficacy against Omicron-KP.3 infection in a mouse model.

## 2. Materials and Methods

### 2.1. SARS-CoV-2 KP3 mRNA Design

KP3 mRNA of SARS-CoV-2 was designed as follows [46,47]. Briefly, a codon-optimized S gene (GISAID accession No. EPI_ISL_18081528) of SARS-CoV-2 Omicron-EG.5 subvariant was used in the study, and its RBD region was replaced by the RBD of the Omicron-KP.3 subvariant of SARS-CoV-2 (hereinafter KP3) (GISAID accession No. EPI_ISL_19130023). This recombinant plasmid has a mutated furin cleavage site and six proline substitutions (i.e., HexaPro sequence) within the S sequence, a signal peptide, tissue plasminogen activator (tPA), at the N-terminus, as well as a trimeric motif sequence (i.e., foldon) and a His_6_ tag sequence at the C-terminus. This recombinant was inserted into a recombinant vector (pCAGGS), and confirmed for correct sequences by Sanger DNA sequencing assay.

### 2.2. SARS-CoV-2 KP3 mRNA Generation

The KP3 mRNA was generated as follows [46]. Briefly, the above recombinant plasmid with correct sequences was linearized and used as the template to generate mRNA based on a Transcription Kit (MEGAscript T7; Thermo Fisher Scientific, Waltham, MA, USA). Pseudo-UTP (Ψ) (APExBIO, Houston, TX, USA), which is a naturally occurring modified nucleoside, was used together with three other nucleosides (ATP, CTP, and GTP) to generate the mRNA, with the goal to enhance the stability of the mRNA. The generated mRNA was purified using RNA Cleanup Kit (New England Biolabs, Ipswich, MA, USA), which was further capped and tailed using Cap 1 Capping System Kit and Poly(A) Polymerase Tailing Kit (CELLSCRIPT, Madison, WI, USA), respectively.

### 2.3. KP3 mRNA Formulation with LNPs and Characterization

The generated KP3 mRNA was subsequently formulated with LNPs [46]. Briefly, the mRNA, which was dissolved in a formulation buffer (PNI; NWW0043, Precision Nanosystems, Vancouver, BC, Canada), was encapsulated with lipids (i.e., GenVoy-ILM; NWW0042, Precision Nanosystems, Vancouver, BC, Canada) at a ratio of 3:1 (mRNA:lipids) to form mRNA-LNPs by NanoAssemblr Ignite Instrument (Precision Nanosystems, Vancouver, BC, Canada). The encapsulated mRNA-LNPs were incubated with PBS and then concentrated at 3000× *g* using a 10 kDa Centrifugal Filter (Amicon Ultra-15; MilliporeSigma, Burlington, MA, USA). The KP3 mRNA-LNPs were tested for efficiency of encapsulation by Invitrogen Quant-iT RiboGreen RNA Assay Kit (Thermo Fisher Scientific, Waltham, MA, USA), reaching about 80%. The mRNA was further tested for endotoxin by LAL Endotoxin Assay Kit (GenScript, Piscataway, NJ, USA), resulting in <1 EU/ml. This encapsulated KP3 mRNA was evaluated for stability and particle sizes by a DLS instrument (i.e., DynaPro NanoStar II Light Scattering Detector) (Wyatt Technology, Santa Barbara, CA, USA).

### 2.4. Flow Cytometry for mRNA Expression

Flow cytometry assay was utilized to assess the relevant proteins encoded by the mRNA [46,47]. Briefly, human 293T cells were split in a 6-well cell culture plate (2 × 10^5^/well) overnight at 37 °C. The KP3 mRNA-LNP (4 µg), or control LNP, was incubated with the cultured cells, followed by addition of Brefeldin A (Biolegend, San Diego, CA, USA) (final concentration: 2.5 µg/ml) 24 h later to block protein secretion. The cells were further cultured for 24 h at 37 °C, centrifuged for 5 min at 200× *g*, and then stained with Fixable Viability Dye eFluor 780 (Thermo Fisher Scientific, Waltham, MA, USA) for 20 min at 4 °C. This step was followed by fixation/permeabilization using Cytofix/Cytoperm kit (BD Biosciences, Franklin Lakes, NJ, USA). The fixed and permeabilized cells were incubated with FITC-mouse-anti-His antibody (Thermo Fisher Scientific, Waltham, MA, USA) or immunized SARS-CoV-2 Omicron S-specific mouse sera (self-prepared polyclonal antibody), followed by goat-anti-mouse IgG-PerCP-eFluor 710 (Thermo Fisher Scientific, Waltham, MA, USA) for 30 min at room temperature. These cells were examined for expression of His or SARS-CoV-2 S protein using a flow cytometer (CytoFLEX) (Beckman Coulter Life Sciences, Brea, CA, USA). FlowJo software (v7.6) was utilized to analyze the results from the flow cytometry assay.

### 2.5. Western Blot for mRNA Expression

Western blot was also utilized to assess the mRNA-encoding, SARS-CoV-2 Omicron S-specific, KP3-RBD target protein. Briefly, after treatment of 293T cells with the KP3 mRNA-LNP or control LNP samples and culture of the cells as described above, RIPA Lysis Buffer (Thermo Fisher Scientific, Waltham, MA, USA) supplied with a protease inhibitor cocktail (Sigma-Aldrich, St. Louis, MO, USA) was utilized to lyse the cells for 30 min on ice, and the cell lysates were centrifuged for 20 min at 14,000× *g*. The supernatant was collected, and the target protein was quantified using the Bradford method (Bio-Rad, Hercules, CA, USA). Equal amounts of total protein from each sample were separated onto an SDS-PAGE gel (8%), which was then transferred to Nitrocellulose membranes (Bio-Rad, Hercules, CA, USA). The non-specific binding on the membranes was blocked by 2% non-fat milk (Bio-Rad, Hercules, CA, USA) for 1 h at room temperature, and the membranes were sequentially incubated with anti-Omicron-S mouse sera overnight at 4 °C, and horseradish peroxidase (HRP)-conjugated goat anti-mouse IgG antibody (Thermo Fisher Scientific, Waltham, MA, USA) for 1 h at room temperature. The signal on the membranes was detected by ECL Chemiluminescent kit (Bio-Rad, Hercules, CA, USA), and the images were captured by an Imaging System instrument (ChemiDoc MP; Bio-Rad, Hercules, CA, USA).

### 2.6. Enzyme-Linked Immunosorbent Assay

KP3 mRNA-induced antibody responses were assessed by an enzyme-linked immunosorbent assay (ELISA) [46]. Briefly, KP.3-RBD-containing S protein (i.e., Omicron-EG.5 S protein whose RBD was replaced by Omicron-KP.3 RBD) (1 µg/ml) was coated onto 96-well, half-area ELISA plates (50 µL/well) overnight at 4 °C. After incubating with blocking buffer for 1 h at 37 °C, the plates were washed with PBST buffer for 5 times, and then incubated with serially diluted mouse sera for 1 h at 37 °C. After additional washes, the plates were incubated with HRP-conjugated anti-mouse IgG-Fab (1:5000, Sigma-Aldrich, St. Louis, MO, USA), anti-mouse IgG1, or anti-mouse IgG2a (1:5000, Thermo Fisher Scientific, Waltham, MA, USA) antibodies for 1 h at 37 °C. The plates were further incubated with 3,3′,5,5′-Tetramethylbenzidine (TMB) substrate (Sigma-Aldrich, St. Louis, MO, USA) and H_2_SO_4_ (1 N), respectively. The 450 nm absorbance values were obtained by Cytation 7 Microplate Multi-Mode Reader and associated Gen5 software (v3.11) (BioTek Instruments, Winooski, VT, USA).

### 2.7. Neutralization Assay Based on SARS-CoV-2 Pseudovirus

Immunized mouse sera were initially assessed for neutralizing antibodies by a SARS-CoV-2 pseudovirus-based neutralization assay [48]. Briefly, recombinant plasmids encoding the following four S proteins of SARS-CoV-2 were constructed, which include the S protein of Omicron-XEC (hereinafter XEC pseudovirus) (GISAID accession No. EPI_ISL_19612171), as well as the S protein of Omicron-EG.5 (GISAID accession No. EPI_ISL_18081528) whose RBD region was replaced by the RBDs of Omicron-KP.2 (hereinafter KP.2 pseudovirus) (GISAID accession No. EPI_ISL_19088331), Omicron-KP.3 (hereinafter KP.3 pseudovirus) (GISAID accession No. EPI_ISL_19130023), and Omicron-NB.1.8.1 (hereinafter NB.1.8.1 pseudovirus) (GISAID accession No. EPI_ISL_19887781), respectively. Each of these four plasmids was co-transfected with two additional plasmids, including pLenti-CMV-luciferase and PS-PAX2 (Addgene, Watertown, MA, USA), into 293T cells using the polyetherimide (Sigma-Aldrich, St. Louis, MO, USA) transfection method. The cell culture medium was changed into fresh DMEM 6–8 h later, and the cells were cultured for 72 h at 37 °C in a cell culture incubator. The pseudovirus-containing cell culture supernatant was then collected for pseudovirus neutralization assay described below. The serially diluted sera from immunized mice were incubated with each pseudovirus for 1 h at 37 °C. The above mixture of sera and pseudovirus was added to 96-well cell culture plates pre-seeded with hACE2/293T cells (i.e., 293T cells expressing SARS-CoV-2 receptor hACE2). After culturing the cells for 24 h at 37 °C, fresh DMEM cell culture medium was added to the plates, and the cells were further cultured for an additional 48 h at 37 °C. Luciferase Cell Culture Lysis Reagent (Promega, Madison, WI, USA) was added to the plates to lyse cells for about 1 h at room temperature. The lysed cells were incubated with Luciferase Assay System substrate (Promega, Madison, WI, USA) and then assessed for luciferase activity using Microplate Multi-Mode Reader (Cytation 7) instrument (BioTek Instruments, Winooski, VT, USA). The serum pseudovirus neutralizing antibody titer was presented as 50% neutralizing antibody titer (NT_50_).

### 2.8. Neutralization Assay Based on Live SARS-CoV-2

Immunized mouse sera were further assessed for neutralizing antibodies by a live SARS-CoV-2-based neutralization assay using a cytopathic effect (CPE) method. Briefly, two Omicron subvariants, namely, KP.2 and KP.3, of live SARS-CoV-2 (100 tissue culture infectious dose 50% (TCID_50_)/well) were, respectively, incubated with mouse sera at serial dilutions for 1 h at 37 °C, and the mixture of sera and virus was added to 96-well cell culture plates pre-seeded with Vero E6 cells. The cells were cultured for 4–5 days at 37 °C and observed for the presence of CPE under a microscope. The neutralizing antibody titer was reported as the highest serum dilution that inhibited at least 50% of the cells from CPE (NT_50_).

### 2.9. Detection of Viral Titers in the Lungs

A standard plaque assay was used to measure viral titers in the lungs of challenge mice [46]. Briefly, lung tissues were collected from SARS-CoV-2-challenged mice, lysates with serial dilutions were added to Vero E6 cells, and the cells were incubated for 1 h at 37 °C. The medium was removed, and the cell culture medium (MEM) supplied with FBS (2%) and carboxymethyl cellulose solution (1.25%) was then added to the cells for continual culture at 37 °C. Four days later, the cultured cells were washed with sterile PBS, followed by fixation with 10% formaldehyde for 2 h at 37 °C, and staining with crystal violet (0.5%). Plaques shown in each well were calculated, and relevant viral titers in lung tissues represented plaque forming unit (PFU/ml) of test tissue samples.

### 2.10. Mice, Immunization, and Sample Collection

Male and female BALB/c mice (17~19 weeks old), BALB/c-background K18-hACE2 transgenic (i.e., BALB/c-hACE2) mice (6~8 weeks old), and C57BL/6-background K18-hACE2 transgenic (i.e., B6-hACE2) mice (10~12 weeks old) were used in this study. These mice were originally purchased from the Jackson Laboratory, and bred and maintained in our animal facilities for the experiments. They were randomly assigned to different groups during the experiments. For immunization schedules, BALB/c and BALB/c-hACE2 mice (5 mice/group) were immunized intradermally (i.d.) with the LNP-encapsulated SARS-CoV-2 KP3 mRNA (10 µg/100 µL/mouse), or LNP control (100 µL/mouse), and boosted twice with the same immunogen at 3-week intervals, which were based on our previously optimal protocols [46,47]. Sera collected 10 days post-third immunization were tested for Omicron-KP.3-specific antibodies using ELISA, as well as for anti-SARS-CoV-2 neutralizing antibodies using pseudovirus and live virus neutralization assays described above.

### 2.11. Challenge of Immunized Mice with SARS-CoV-2 Omicron Subvariant

KP3 mRNA-immunized mice were challenged with an Omicron subvariant of SARS-CoV-2 [48]. Briefly, about 2 months after the third immunization of KP3 mRNA, BALB/c-hACE2 mice were intranasally (i.n.) challenged with the Omicron-KP.3 subvariant of SARS-CoV-2 (10^4^ PFU/mouse). Five days later, lungs collected from the challenged mice were assessed for viral titers by plaque assay, as described above.

### 2.12. Challenge of Naïve Mice Receiving Transferred Immune Sera

Naïve mice receiving immunized mouse sera were challenged with Omicron-KP.3 subvariant of SARS-CoV-2 [48]. Briefly, about 5 months after the third immunization of KP3 mRNA, BALB/c mice were further boosted with the same immunogen (LNP-encapsulated KP3 mRNA, 10 µg/100 µL/mouse), or LNP control (100 µL/mouse). Then, 10–20 days later, sera (about 100 µL/mouse or more in total) were collected 3 times through facial bleeding. After heat inactivation for 30 min at 56 °C, the pooled sera (200 µL/mouse) from each group were intraperitoneally (i.p.) injected into naïve B6-hACE2-transgenic mice (5 mice/group). After 6 h, these mice were infected (i.n.) with Omicron-KP.3 subvariant of SARS-CoV-2 (10^4^ PFU/mouse), and 5 days later, lungs were collected from the challenged mice and assessed for viral titers by plaque assay described above. The pooled sera were also detected for Omicron-S-specific IgG antibody titer by ELISA, as well as neutralizing antibody titer by pseudovirus and live virus neutralization assays against SARS-CoV-2 Omicorn-KP.3 subvariant, as described above.

### 2.13. Statistical Analysis

The experimental results were assessed for statistical differences using GraphPad Prism 10 statistical software. Tukey’s multiple comparison test (under Ordinary one-way ANOVA) was applied to compare statistical significance among different groups, and an unpaired Student’s *t* test was applied to compare statistical significance between two groups. *p* < 0.05, *p* < 0.01, and *p* < 0.001 are represented by *, **, and ***, respectively, and ns represents no significant difference among different groups.

## 3. Results

### 3.1. Construction and Characterization of the SARS-CoV-2 KP.3 RBD-Containing Omicron-S mRNA Vaccine

**Figure 1:**
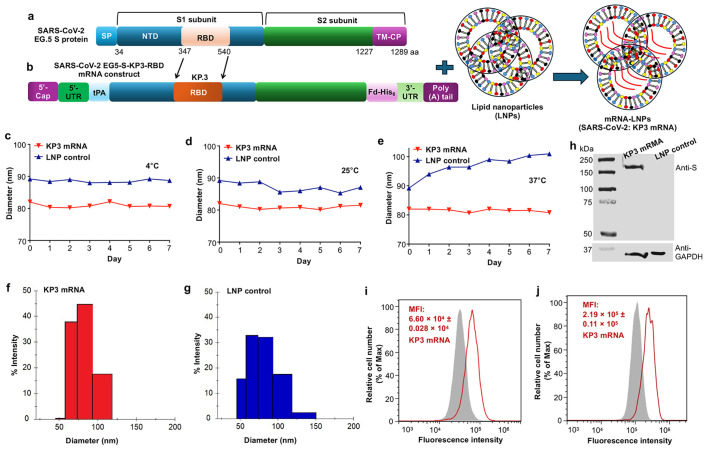
KP3-mRNA construct and characterization. (**a**) Schematic figure of spike (S) protein of SARS-CoV-2 EG.5 Omicron subvariant. SP, NTD, and RBD represent signal peptide, N-terminal domain, and receptor-binding domain, respectively. TM-CP represents transmembrane and cytoplasmic domains. Amino acid (aa) residues for related regions are shown. (**b**) Schematic figure of SARS-CoV-2 EG5-S-KP3-RBD mRNA (i.e., KP3-mRNA) with a C-terminal foldon (Fd) trimeric motif and a His_6_ tag. This mRNA was encapsulated with lipid nanoparticles (LNPs) constituting mRNA-LNPs. 5′-UTR and 3′-UTR, untranslated regions. tPA, tissue plasminogen activator signal peptide. Stability of the LNP-formulated KP3 mRNA or LNP control measured by DynaPro NanoStar II Light Scattering Detector (DLS). The mRNA and control samples were stored for up to 7 days at 4 °C (**c**), 25 °C (**d**), and 37 °C (**e**), respectively, and then measured for the particle sizes (diameter: nm) by the DLS instrument. Histogram maps of the particle sizes of LNP-formulated KP3 mRNA (**f**) or LNP control (**g**) measured by the DLS. (**h**) Expression of Omicron-S mRNA-encoding protein by Western blot analysis. The top side indicates SARS-CoV-2-specific anti-Omicron-S mouse sera; the bottom side indicates anti-GAPDH antibody as an internal control. Protein molecular weight marker (kDa) is shown on the left. (**i**,**j**) Representative figures showing expression of His-tagged, KP3 mRNA-encoding proteins by flow cytometry analysis. The LNP-formulated KP3 mRNA or LNP control samples were added to 293T cells, whereas the cells were cultured for 48 h at 37 °C, and then incubated with FITC-mouse-anti-His antibody (**i**), or anti-Omicron-S mouse sera, followed by goat-anti-mouse IgG-PerCP-eFluor 710 antibody (**j**), which were further analyzed for fluorescence intensity by flow cytometry. The shaded gray area represents control cells incubated with LNP only, and the red line represents target cells incubated with LNP-formulated KP3 mRNA. MFI refers to median fluorescence intensity.

SARS-CoV-2 KP.3 RBD-containing Omicron-S mRNA (referred to hereafter as KP3 mRNA) was constructed by inserting the RBD of Omicron-KP.3 into the S backbone of Omicron-EG.5 (Figure 1a,b), which also contains a 5′-terminal tPA signal peptide, a 3′-foldon trimeric motif, and a His_6_ tag, in addition to the encoded HexaPro sequence. The foldon trimeric sequence would facilitate the expressed protein to form a native trimeric structure, and a tPA signal peptide would help direct the secretion of the expressed protein [48,49]. KP3 mRNA was synthesized using the MEGAscript T7 Transcription Kit (Thermo Fisher Scientific, Waltham, MA, USA) and regular nucleosides ATP, CTP, and GTP, plus a naturally occurring modified nucleoside, Pseudo-UTP; the latter is used to eliminate mRNA-associated innate immune activation and improve the stability of the synthesized mRNA. The mRNA also contained a Cap sequence and an untranslated region (5′-UTR) at the 5′ terminus, as well as a 3′-UTR and a ploy(A) tail at the 3′ terminus (Figure 1b). The synthesized KP3 mRNA was encapsulated within LNPs to form mRNA-LNPs (Figure 1b), which were then used for characterization and immunization. DLS analysis showed that the LNP-encapsulated KP3 mRNA was stable, maintaining a similar size (~80–82 nm in diameter) when kept for up to seven days at 4 °C, 25 °C, or 37 °C (Figure 1c–e). By contrast, LNPs alone were slightly larger and relatively unstable than LNP-encapsulated KP3 mRNA (Figure 1c–e). Similar results were also revealed in the histogram figures measured by the DLS instrument (Figure 1f,g). LNP-encapsulated KP3 mRNA effectively expressed the encoded protein, which was detected by Western blot using anti-Omicron-S mouse sera (polyclonal antibody) against SARS-CoV-2 (Figure 1h), as well as by flow cytometry using a FITC-anti-His antibody (Figure 1i) and SARS-CoV-2-specific anti-Omicron-S mouse sera (Figure 1j). These results demonstrate that the LNP-encapsulated KP3 mRNA maintains high stability under different conditions and expresses the target proteins effectively.

### 3.2. The SARS-CoV-2 KP3 mRNA Vaccine Induces Potent and Specific Antibody Responses

**Figure 2:**
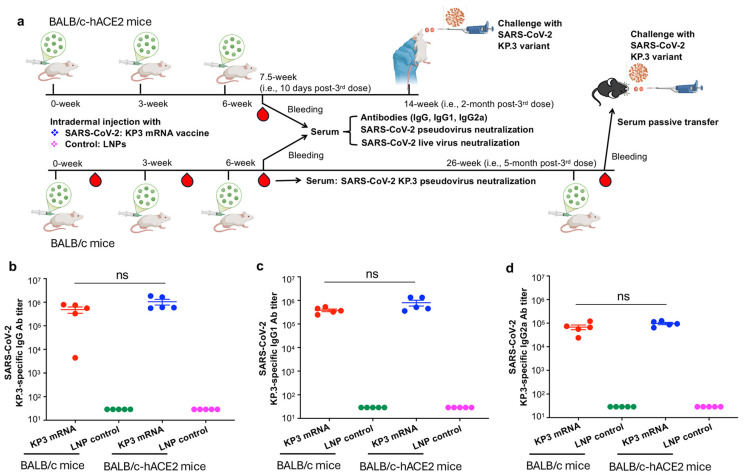
Immunization and challenge schedules and resulting antibody responses. (**a**) Immunization schedule. BALB/c or BALB/c-hACE2 mice were immunized with LNP-encapsulated SARS-CoV-2 KP3-mRNA vaccine or LNP control for three times at 3-week intervals, and mouse sera were collected at 7.5 weeks (i.e., 10 days after the third immunization) for detection of antibody responses by ELISA and neutralizing antibodies by pseudovirus and live virus neutralization assays, respectively. Sera collected from BALB/c mice at 10 days post each immunization were also compared for neutralizing antibodies against KP.3 pseudovirus infection. Two months after the third dose, immunized BALB/c-hACE2 mice were infected (i.n.) with Omicron-KP.3 subvariant of SARS-CoV-2 at 10^4^ PFU (plaque-forming unit) per mouse. Five days after virus infection, the lungs of mice were collected, and viral titers were measured by plaque assay. Five months after the third dose, immunized BALB/c mice were boosted again, and sera were collected 3 times 10–20 days later, which were injected (i.p.) into naïve B6-hACE2 mice, and then measured for viral titers in the lungs as described above. Measurement of SARS-CoV-2 KP.3-specific IgG (**b**), IgG1 (**c**), or IgG2a (**d**) antibody (Ab) by ELISA for sera collected at 7.5 weeks (i.e., 10 days after the third immunization). ELISA plates were pre-coated with KP.3 RBD-containing Omicron-S protein of SARS-CoV-2, and the results represent the mean ± s.e.m (i.e., standard deviation of the mean) of five mice per group. Statistical significance among various groups was analyzed by Tukey’s multiple comparison test (under Ordinary one-way ANOVA). ns, no significant difference among the tested groups. The experiments were repeated once, and showed similar results.

To evaluate whether LNP-encapsulated KP3 mRNA induces specific antibody responses, we immunized BALB/c mice three times, collected serum samples after the third immunization, and measured KP.3-specific IgG and subtype antibodies (IgG1 and IgG2a) (Figure 2a). BALB/c-based transgenic mice expressing SARS-CoV-2 receptor hACE2 (BALB/c-hACE2) were also immunized similarly with KP3 mRNA, after which these antibodies were again measured, since these mice were used for the subsequent virus challenge study (Figure 2a). As expected, KP3 mRNA elicited potent Omicron-KP.3 RBD-specific IgG antibodies in both BALB/c mice and BALB/c-based hACE2 transgenic mice (Figure 2b). There was no significant difference in the titers of KP.3-RBD-specific IgG, IgG1 subtype antibodies, or IgG2a subtype antibodies between the two mouse strains (Figure 2b–d). By contrast, the LNP control (without mRNA) induced only background levels of IgG, as well as IgG1 and IgG2a subtype antibodies (Figure 2b–d). These results demonstrate that the LNP-encapsulated KP3 mRNA is highly immunogenic and elicits effective antibody responses specific for the target protein.

### 3.3. The SARS-CoV-2 KP3 mRNA Vaccine Induces Broad-Spectrum and Potent Neutralizing Antibodies Against Multiple Omicron Subvariants

**Figure 3:**
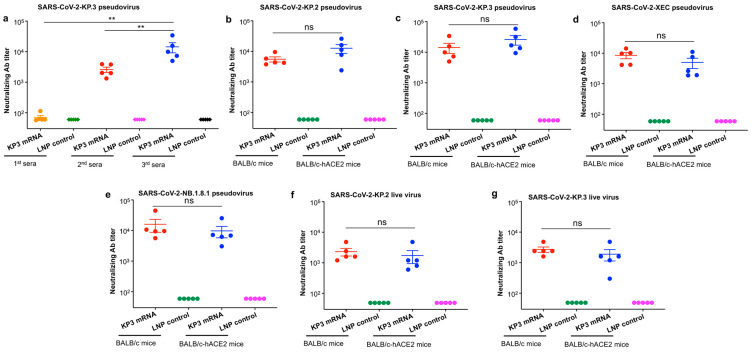
KP3 mRNA encapsulated with LNPs induced neutralizing antibodies against multiple SARS-CoV-2 Omicron subvariants. (**a**) Sera were collected from immunized BALB/c mice 10 days after the first, second, and third doses, and assessed for neutralizing antibody (Ab) titer against Omicron-KP.3 pseudovirus of SARS-CoV-2. Sera collected from BALB/c or BALB/c-hACE2 mice at 7.5 weeks (i.e., 10 days after the third immunization) were assessed for neutralizing Ab titer against Omicron-KP.2 (**b**), Omicron-KP.3 (**c**), Omicron-XEC (**d**), and Omicron-NB.1.8.1 (**e**) pseudoviruses of SARS-CoV-2 by pseudovirus neutralization assay, as well as against live Omicron-KP.2 (**f**) or live Omicron-KP.3 (**g**) of SARS-CoV-2 by CPE-based live virus neutralization assay. The 50% neutralizing antibody titer (NT_50_) is presented as the mean ± s.e.m of five mice per group. Statistical significance among various groups was analyzed by Tukey’s multiple comparison test (under Ordinary one-way ANOVA). ** and ns represent *p* < 0.01 and no significant difference, respectively, among the tested groups. The experiments were repeated once, obtaining similar results.

To initially evaluate and compare neutralizing antibodies induced by LNP-encapsulated KP3 mRNA after each immunization dosage, we collected sera from immunized BALB/c mice 10 days after each dose and tested for their neutralizing antibodies against pseudotyped Omicron-KP.3 subvariant (Figure 2a). The results showed that the third-dose immunization of KP3 mRNA induced the highest neutralizing antibody titer, which was significantly higher than that induced by the first and second doses of immunization (Figure 3a). This data indicates that a three-dose immunization protocol is optimal for KP3 mRNA to elicit high-titer neutralizing antibodies against SARS-CoV-2.

To further evaluate the ability of LNP-encapsulated KP3 mRNA to induce broadly neutralizing antibodies, we used sera of both BALB/c mice and BALB/c-based hACE2 transgenic mice from 10 days after the third immunization, and tested their neutralizing activity against multiple pseudotyped and live SARS-CoV-2 Omicron subvariants (Figure 2a). In general, antibodies induced by KP3 mRNA neutralized all of the recent SARS-CoV-2 pseudotyped Omicron subvariants tested, including KP.2, KP.3, XEC, and NB.1.8.1, with similarly high levels being elicited in both BALB/c mice and BALB/c-based hACE2 transgenic mice (the difference was not significant) (Figure 3b–e). KP3 mRNA also induced effective neutralizing antibodies specific for recent live SARS-CoV-2 Omicron subvariants, including KP.2 and KP.3, again with no significant difference between the two mouse strains (Figure 3f,g). By contrast, the LNP control without mRNA induced only background levels of neutralizing antibodies in test mice (Figure 3). These results demonstrate that the LNP-encapsulated KP3 mRNA is capable of inducing broad and potent neutralizing antibody responses against multiple subvariants of SARS-CoV-2 Omicron.

### 3.4. The SARS-CoV-2 KP3 mRNA Vaccine Protects Mice from Subsequent Challenge with SARS-CoV-2 Omicron-KP.3

**Figure 4:**
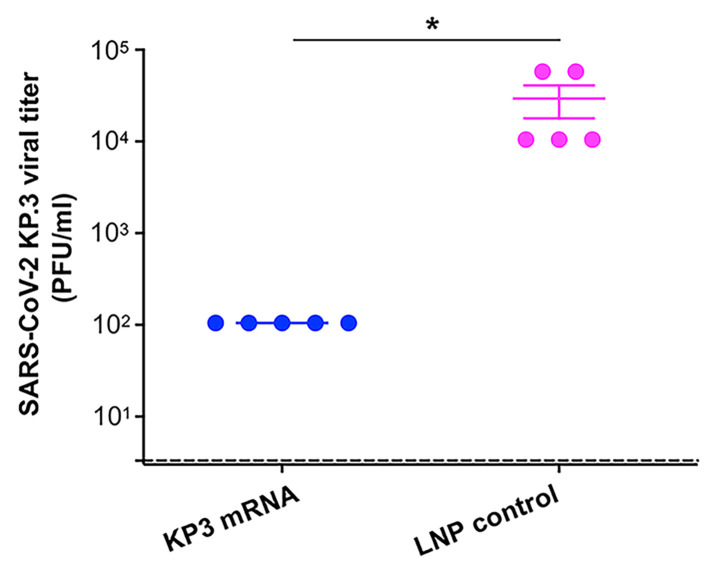
LNP-encapsulated KP3 mRNA protected immunized mice from SARS-CoV-2 Omicron variant challenge. Two months after the third immunization of LNP-encapsulated KP3 mRNA or LNP control, BALB/c-hACE2 mice were challenged (i.n.) with SARS-CoV-2 Omicron-KP.3 subvariant, and 5 days later, they were sacrificed for measurement of viral titers in the lungs by plaque assay. The data was calculated as PFU/ml of viral titers and is expressed as the mean ± s.e.m of five mice per group. The detection limit (dotted line) was 3.3 PFU/ml. Unpaired Student’s *t* test was utilized to analyze statistical significance (*: *p* < 0.05) between the two groups. The experiments were repeated once, and showed similar results.

To assess whether the KP3 mRNA encapsulated with LNPs may protect against challenge with SARS-CoV-2, vaccinated BALB/c-hACE2 transgenic mice were infected (i.n.) with the Omicron-KP.3 subvariant of SARS-CoV-2 two months after the final immunization (Figure 2a). Five days later, the challenged mice were sacrificed, the lungs were collected, and their viral titers were assessed by a plaque assay (Figure 2a). KP3 mRNA protected mice against Omicron-KP.3 challenge, and the viral titer in the lungs of mice immunized with the KP3 mRNA was significantly lower than the viral titer in the lungs of mice inoculated with the control, LNPs alone (Figure 4). These results indicate that the KP3 mRNA encapsulated with LNPs protects immunized mice effectively from subsequent challenge with Omicron-KP.3 subvariant of SARS-CoV-2.

### 3.5. SARS-CoV-2 KP3 mRNA Immune Sera Show Passive Protective Efficacy Against Challenge with SARS-CoV-2 Omicron-KP.3

**Figure 5:**
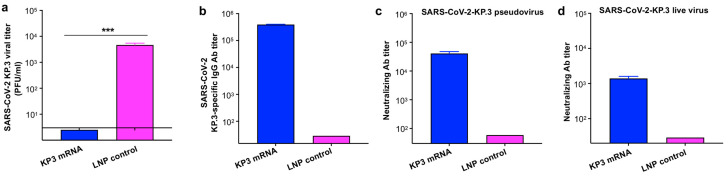
Immune sera from mice vaccinated with LNP-encapsulated KP3 mRNA passively protected naïve mice from challenge of Omicron variant of SARS-CoV-2. (**a**) SARS-CoV-2 titer in the lungs of challenged mice. Sera were collected 3 times from KP3 mRNA-immunized mice or control mice 10–20 days post-fourth immunization, and pooled sera from each group were transferred (i.p.) into naïve B6-hACE2 mice. Then 6 h later, these mice were infected (i.n.) with Omicron-KP.3 subvariant of SARS-CoV-2. Five days after, lungs were collected from the challenged mice and assessed for viral titers by plaque assay. The viral titer is shown as PFU/ml, and the results are presented as the mean ± s.e.m of five mice per group. A total of 3.3 PFU/ml was used for the detection limit. Statistical significance (***: *p* < 0.001) between the two groups was analyzed using unpaired Student’s *t* test. (**b**) ELISA for measurement of the pooled sera for IgG antibody (Ab) titer specific to SARS-CoV-2 KP.3 protein. ELISA plates were pre-coated with KP.3 RBD-containing Omicron-S protein of SARS-CoV-2. (**c**) Pseudovirus neutralization assay was used to measure the pooled sera for neutralizing Ab titer (NT_50_) against SARS-CoV-2 Omicron-KP.3 pseudovirus. (**d**) CPE-based live virus neutralization assay was used to measure the pooled sera for neutralizing Ab titer (NT_50_) against authentic Omicron-KP.3 of SARS-CoV-2. The results (in **b**–**d**) are presented as the mean ± s.e.m of duplicate or quadruple wells of pooled serum samples. The experiments were repeated once, which yielded similar results.

To evaluate whether neutralizing antibodies induced by LNP-encapsulated KP3 mRNA vaccine play a role in protection against SARS-CoV-2 challenge, and whether they protect other mouse strains, we boosted immunized BALB/c mice at five months after the third dose of vaccine. Sera were collected and pooled prior to injection (i.p.) into B6-hACE2 mice, which were then challenged 6 h later with SARS-CoV-2 Omicron-KP.3, and viral titers in the lungs of the challenged mice were measured five days post-challenge (Figure 2a). Viral titers in the lungs of mice receiving KP3-mRNA immune sera were below the limit of detection, and significantly lower than those in control mice receiving LNP control immune sera (Figure 5a). Notably, sera pooled from KP3 mRNA-immunized mice contained high affinity antibodies specific for the SARS-CoV-2 KP.3-RBD-containing protein, as measured by ELISA (Figure 5b), leading to potent neutralization of both the Omicron-KP.3 pseudovirus and authentic virus (Figure 5c,d). The above data indicates that KP3 mRNA provides passive protection against SARS-CoV-2 Omicron-KP.3 infection and that the protection positively associates with serum neutralizing antibodies.

## 4. Discussion

Since COVID-19 first emerged in 2019, the genome encoding the S protein of SARS-CoV-2, particularly the RBD region, has undergone marked and continuous changes, resulting in the presence of five VOC strains to date [21,22,50]. These variants have enabled the virus to easily elude immune responses generated by previous infection or vaccination [51,52,53,54], calling for a continual need to develop effective vaccines against SARS-CoV-2 variants.

The Omicron variant, which first emerged in late December of 2021, has evolved to yield numerous subvariants [23,55,56,57,58]. Omicron harbors at least 30 amino acid residue mutations within the full-length S protein, more than 15 of which are in the RBD region alone, compared with the original parent virus [25,56,59,60]. However, the backbone sequence of the S protein is relevantly conserved across different Omicron strains, and even across early variants [48], raising hopes of developing a universal vaccine that targets the Omicron variant and subvariants.

In addition to the two SARS-CoV-2 mRNA vaccines approved for human use, other more versatile mRNA vaccines against SARS-CoV-2, which are based on self-amplification or nucleoside modifications, have been developed for preclinical and clinical use [61,62,63,64,65]. The majority of these vaccines target the S protein; however, most are based on S proteins carrying the RBDs from the same strain and/or variant, potentially resulting in reduced neutralizing activity against heterologous variants or subvariants [66,67,68]. To improve the immunogenicity and/or protection provided by mRNA vaccines against SARS-CoV-2 variants, dual or bivalent mRNA vaccination strategies, such as combining an S-encoding mRNA with an N-encoding mRNA, or co-delivering two mRNAs encoding the S proteins from different variants/subvariants, have been devised [64,69,70,71,72].

Different from the above approaches, we designed a unique mRNA vaccine comprising the Omicron-KP.3 RBD and the RBD-truncated S backbone of the earlier Omicron subvariant EG.5, with the goal to induce broadly neutralizing antibodies against homologous Omicron subvariant, as well as heterologous Omicron subvariants, due to the sequence conservation of the S backbone region. It should be mentioned that vaccines encoding a variant RBD based on the conserved backbone region of the S protein of SARS-CoV-2 have a greater chance of maintaining the conformational structure and, therefore, the strong immunogenicity of the S protein, thereby resulting in much improved neutralizing activity against different variants or subvariants [46,48,49].

It is worth noting that naked mRNAs are not stable and often fail to enter target cells efficiently; therefore, they require effective delivery tools, such as LNPs that facilitate delivery of mRNA to the cell cytoplasm prior to subsequent protein expression [73]. It has been shown that mRNA-LNPs with particle sizes of 80–100 nm exhibit a low degree of degradation and present long-term stability and increased immunogenicity [74]. Here, the designed mRNA vaccine was encapsulated with LNPs for in vitro and in vivo delivery. Indeed, we found that LNP-formulated KP3 mRNA vaccine (with particle size of 80–82 nm) led to strong stability when stored at different temperatures for up to seven days, and that the target proteins were expressed strongly. The chimeric mRNA vaccine was further examined for its broadly neutralizing activity and effective protective efficacy. As expected, this nucleoside-modified, LNP-encapsulated KP3 mRNA vaccine elicited broad and potent neutralizing antibodies not only against homologous subvariants containing the RBD of Omicron-KP.3, but also against other more recent Omicron subvariants such as KP.2, XEC, and NB.1.8.1. Of note, the mRNA vaccine also protected immunized mice against infection with Omicron-KP.3, and this protective efficacy was associated positively with the titer of serum neutralizing antibodies.

Immune imprinting phenomenon has been notified in SARS-CoV-2 mRNA vaccines, in which recall antibodies against the ancestral S-encoding vaccines affect subsequent immune responses induced by bivalent vaccines encoding S proteins of both ancestral and Omicron BA.4-5 subvariants or monovalent XBB.1.5 mRNA vaccine [75]. Persistent immune imprinting was also found in humans receiving booster dose(s) of mRNA vaccine encoding XBB.1.5 S protein, where neutralizing antibodies against the current variant cross-reacted with the previous ancestral S, recalling ancestral RBD-specific, pre-existing memory B cells [76]. Such immune imprinting phenomenon would not be expected in the present study, since the designed vaccine was immunized into naïve mice, and boosts were performed using the same immunogen. Future studies would be preferable to evaluate potential immune imprinting of the vaccine after additional dose(s) with the same or different immunogen(s) (i.e., variant or ancestral strain), followed by investigating the production of antigen-specific humoral or memory immune responses targeting variant or ancestral S protein.

## 5. Conclusions

Taken together, the data presented herein suggest that the designed and well-characterized, nucleoside-modified KP3 mRNA vaccine has potential for further development as a pan-Omicron vaccine that protects against current and future Omicron subvariants.

## Data Availability

All relevant data is reported in this study.

## Abbreviations

The following abbreviations are used in this manuscript:

ACE2	Angiotensin-converting enzyme 2
COVID-19	Coronavirus Disease 2019
CPE	Cytopathic effect
ELISA	Enzyme-linked immunosorbent assay
i.d.	Intradermal
i.n.	Intranasal
i.p.	Intraperitoneal
LNP	lipid nanoparticle
MERS-CoV	Middle East respiratory syndrome coronavirus
NT_50_	50% neutralizing antibody titer
PFU	Plaque-forming unit
RBD	Receptor-binding domain
S	Spike
SARS-CoV-2	Severe acute respiratory syndrome coronavirus 2
UTR	Untranslated region
VOCs	Variants of concern
